# Bis(dicyclo­hexyl­ammonium) μ-oxalato-κ^4^ *O* ^1^,*O* ^2^:*O* ^1′^,*O* ^2′^-bis­[aqua­(oxalato-κ^2^ *O* ^1^,*O* ^2^)diphenyl­stannate(IV)]

**DOI:** 10.1107/S1600536810046738

**Published:** 2010-11-24

**Authors:** Ndongo Gueye, Libasse Diop, K. C. Kieran Molloy, Gabrielle Kociok-Köhn

**Affiliations:** a Laboratoire de Chimie Minérale et Analytique, Département de Chimie, Faculté des Sciences et Techniques, Université Cheikh Anta Diop, Dakar, Senegal; b Department of Chemistry, University of Bath, Claverton Down, Bath BA2 7AY, England

## Abstract

The structure of the title compound, (C_12_H_24_N)_2_[Sn_2_(C_6_H_5_)_4_(C_2_O_4_)_3_(H_2_O)_2_], consists of a bischelating oxalate ion, located on an inversion center, which is linked to two SnPh_2_ groups. The coordination sphere of the Sn(IV) ion is completed by a monochelating oxalate anion and a water mol­ecule. The Sn(IV) atoms are thus seven-coordinated. The discrete binuclear units are further connected by hydrogen bonds, leading to a supra­molecular crystal structure. The asymmetric unit contains one half dianion and one (Cy_2_NH_2_)^+^ cation.

## Related literature

For background to organotin(IV) chemistry, see: Ballmann *et al.* (2009 ▶); Diallo *et al.* (2007 ▶); Diassé-Sarr *et al.* (1997 ▶); Ng *et al.* (1992 ▶); Singh *et al.* (2008 ▶); de Sousa *et al.* (2007 ▶); Wang *et al.* (2009 ▶); Xanthopoulou *et al.* (2007 ▶, 2008 ▶); Zia-ur-Rahman *et al.* (2007 ▶). For related Sn(IV) structures, see: Diop *et al.* (2002 ▶, 2003 ▶).
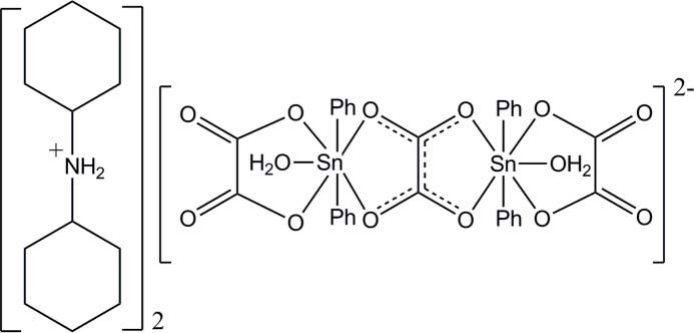


## Experimental

### 

#### Crystal data

(C_12_H_24_N)_2_[Sn_2_(C_6_H_5_)_4_(C_2_O_4_)_3_(H_2_O)_2_]
*M* *_r_* = 1210.52
Monoclinic, 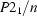

*a* = 13.1725 (4) Å
*b* = 14.6121 (4) Å
*c* = 14.1139 (4) Å
β = 100.869 (2)°
*V* = 2667.88 (13) Å^3^
*Z* = 2
Mo *K*α radiation
μ = 1.00 mm^−1^
*T* = 150 K
0.2 × 0.2 × 0.2 mm

#### Data collection

Nonius KappaCCD diffractometer
Absorption correction: multi-scan (*SORTAV*; Blessing, 1995 ▶) *T*_min_ = 0.825, *T*_max_ = 0.825
48411 measured reflections
6114 independent reflections
4069 reflections with *I* > 2σ(*I*)
*R*_int_ = 0.126

#### Refinement

*R*[*F* ^2^ > 2σ(*F* ^2^)] = 0.040
*wR*(*F* ^2^) = 0.082
*S* = 1.01
6114 reflections
341 parameters
2 restraints
H atoms treated by a mixture of independent and constrained refinement
Δρ_max_ = 0.54 e Å^−3^
Δρ_min_ = −0.72 e Å^−3^

### 

Data collection: *COLLECT* (Nonius, 2000 ▶); cell refinement: *SCALEPACK* (Otwinowski & Minor, 1997 ▶); data reduction: *DENZO* (Otwinowski & Minor, 1997 ▶) and *SCALEPACK*; program(s) used to solve structure: *SIR97* (Altomare *et al.*, 1999 ▶); program(s) used to refine structure: *SHELXL97* (Sheldrick, 2008 ▶); molecular graphics: *ORTEP-3* (Farrugia, 1997 ▶); software used to prepare material for publication: *SHELXL97*.

## Supplementary Material

Crystal structure: contains datablocks I, global. DOI: 10.1107/S1600536810046738/bh2310sup1.cif

Structure factors: contains datablocks I. DOI: 10.1107/S1600536810046738/bh2310Isup2.hkl

Additional supplementary materials:  crystallographic information; 3D view; checkCIF report

## Figures and Tables

**Table 1:** Hydrogen-bond geometry (Å, °)

*D*—H⋯*A*	*D*—H	H⋯*A*	*D*⋯*A*	*D*—H⋯*A*
O7—H7*B*⋯O4^i^	0.90 (4)	1.77 (4)	2.663 (3)	175 (4)
N—H1*A*⋯O3^ii^	0.84 (4)	2.12 (3)	2.910 (4)	155 (3)
N—H1*A*⋯O4^ii^	0.84 (4)	2.37 (4)	2.986 (4)	130 (3)
N—H1*B*⋯O6^iii^	0.91 (4)	2.08 (4)	2.960 (4)	164 (4)

Symmetry codes: (i) 
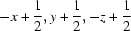
; (ii) 
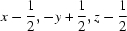
; (iii) 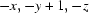.

**Fig. 1.**
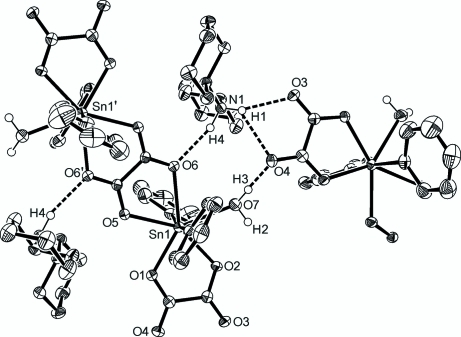
A part of the crystal structure of the title compound, with the atom-numbering scheme. Displacement ellipsoids are drawn at the 50% probability level. Dashed bonds represent hydrogen bonds.

## supplementary crystallographic information

### Comment

In the dynamic of our research work on organotin(IV) chemistry (Diallo *et al.*, 2007; Diassé-Sarr *et al.*, 1997) because of several applications found (Xanthopoulou *et al.*, 2007, 2008; Zia-ur-Rahman *et al.*, 2007; Singh *et al.*, 2008; Wang *et al.*, 2009; Ballmann *et al.*, 2009; de Sousa *et al.*, 2007) and our interest in the coordinating behaviour of oxyanions in this family of compounds, we had yet reported the crystal structures of C_2_O_4_(SnPh_3_)_2_ (Diop *et al.*, 2003) and SO_4_(SnPh_3_)_2_.H_2_O (Diop *et al.*, 2002) and have initiated here the study of the interactions between (Cy_2_NH_2_)_2_C_2_O_4_.2H_2_O and C_2_O_4_(SnPh_3_)_2_ which has yielded the studied compound.

The asymmetric unit consists of one half of the molecule, located about an inversion centre at the mid-point of the C3—C3^i^ bond (symmetry code i: -*x*, 1 - *y*, -*z*). In its units structure two SnPh_2_ residues are linked by a central bichelating oxalate ion [O_5_O_6_:*O*_5_O_6_] and every SnPh_2_ residue is then linked to another monochelating anion [O1, O2]. A water molecule completes the tin centre coordination to seven, which can be described as a distorted *trans*-C_2_SnO_5_ pentagonal bipyramidal geometry [C—Sn—C angle: 168.75 (13)°]. Within the bridging carboxylate all the C—O bonds are equal within experimental error, implying complete delocalization of double-bond character within this residue. The bond lengths C1—O1 and C2—O2, [1.273 (4) Å], and C2—O3, C1—O4 [1.225 (4) and 1.247 (4) Å] indicate respectively a single and double bond character; the bond length C1—O4 results from involvement of O4 in two distinct hydrogen bonds. Among the Sn—O bonds, Sn1—O6 is notably longer, O6 being the only oxygen of this kind involved in hydrogen bonding.

Every moiety is then connected to its neighbour by three types of hydrogen bonds: one O—H···O type involving an H atom of the water molecule and one O atom of monochelating oxalate [O7—H7B···O4], one N—H···O contact involving one O atom of the bichelating oxalate anion and the cation [N—H1B···O6] and a third bifurcated one also involving the cation [N—H1A···O3 and N—H1A···O4], giving a supramolecular crystal structure.

A similar structure, bearing butyl groups in place of phenyl, was previously reported (Ng *et al.*, 1992).

### Experimental

Crystals of the title compound were obtained by allowing (Cy_2_NH_2_)_2_C_2_O_4_.2H_2_O (90 mmol in 15 ml e thanol) to react with C_2_O_4_(SnPh_3_)_2_ (45 mmol in 15 ml e thanol). The mixture was stirred during several hours and slow solvent evaporation afforded crystals suitable for X-rays studies.

### Refinement

All C–bonded H atoms were placed in idealized positions (C—H in the range 0.95 to 1.00 Å), while H atoms bonded to N and O atoms were considered as free atoms. Isotropic displacement parameters for H atoms were calculated from *U*_eq_ of their parent atoms.

### Crystal data

**Table:**

(C_12_H_24_N)_2_[Sn_2_(C_6_H_5_)_4_(C_2_O_4_)_3_(H_2_O)_2_]	*F*(000) = 1244
*M**_r_* = 1210.52	*D*_x_ = 1.507 Mg m^−^^3^
Monoclinic, *P*2_1_/*n*	Mo *K*α radiation, λ = 0.71073 Å
Hall symbol: -P 2yn	Cell parameters from 29450 reflections
*a* = 13.1725 (4) Å	θ = 2.9–27.5°
*b* = 14.6121 (4) Å	µ = 1.00 mm^−^^1^
*c* = 14.1139 (4) Å	*T* = 150 K
β = 100.869 (2)°	Irregular, colourless
*V* = 2667.88 (13) Å^3^	0.2 × 0.2 × 0.2 mm
*Z* = 2	

### Data collection

**Table:**

Nonius KappaCCD diffractometer	6114 independent reflections
Radiation source: fine-focus sealed tube	4069 reflections with *I* > 2σ(*I*)
graphite	*R*_int_ = 0.126
293 2.0 degree images with φ and ω scans	θ_max_ = 27.5°, θ_min_ = 4.1°
Absorption correction: multi-scan (*SORTAV*;Blessing, 1995)	*h* = −17→17
*T*_min_ = 0.825, *T*_max_ = 0.825	*k* = −18→18
48411 measured reflections	*l* = −18→18

### Refinement

**Table:**

Refinement on *F*^2^	Primary atom site location: structure-invariant direct methods
Least-squares matrix: full	Secondary atom site location: difference Fourier map
*R*[*F*^2^ > 2σ(*F*^2^)] = 0.040	Hydrogen site location: inferred from neighbouring sites
*wR*(*F*^2^) = 0.082	H atoms treated by a mixture of independent and constrained refinement
*S* = 1.01	*w* = 1/[σ^2^(*F*_o_^2^) + (0.0335*P*)^2^ + 0.6086*P*] where *P* = (*F*_o_^2^ + 2*F*_c_^2^)/3
6114 reflections	(Δ/σ)_max_ = 0.001
341 parameters	Δρ_max_ = 0.54 e Å^−^^3^
2 restraints	Δρ_min_ = −0.72 e Å^−^^3^
0 constraints	

### Fractional atomic coordinates and isotropic or equivalent isotropic displacement parameters (Å^2^)

**Table:**

	*x*	*y*	*z*	*U*_iso_*/*U*_eq_	
Sn	0.208644 (18)	0.515508 (15)	0.133115 (17)	0.02034 (8)	
O1	0.27245 (18)	0.37817 (14)	0.16466 (16)	0.0235 (5)	
O2	0.35282 (17)	0.53257 (14)	0.24319 (16)	0.0242 (5)	
O3	0.47106 (18)	0.45574 (15)	0.34724 (17)	0.0285 (6)	
O4	0.38762 (18)	0.29623 (15)	0.26721 (17)	0.0275 (6)	
O5	0.09563 (17)	0.41884 (14)	0.04184 (16)	0.0230 (5)	
O6	0.06436 (17)	0.60103 (14)	0.04207 (15)	0.0210 (5)	
O7	0.2413 (2)	0.67274 (16)	0.18266 (19)	0.0292 (6)	
H7A	0.292 (2)	0.669 (3)	0.233 (2)	0.052 (14)*	
H7B	0.197 (3)	0.712 (3)	0.202 (3)	0.074 (16)*	
N	−0.0015 (2)	0.2042 (2)	−0.0277 (2)	0.0214 (6)	
H1A	−0.019 (3)	0.170 (2)	−0.076 (3)	0.027 (10)*	
H1B	−0.027 (3)	0.261 (3)	−0.044 (3)	0.038 (11)*	
C1	0.3500 (3)	0.3702 (2)	0.2332 (2)	0.0216 (8)	
C2	0.3979 (3)	0.4602 (2)	0.2795 (2)	0.0211 (7)	
C3	0.0088 (3)	0.4480 (2)	−0.0001 (2)	0.0196 (7)	
C4	0.1110 (3)	0.5052 (2)	0.2372 (2)	0.0225 (7)	
C5	0.0459 (3)	0.5767 (2)	0.2523 (3)	0.0277 (8)	
H5	0.0467	0.6321	0.2172	0.033*	
C6	−0.0202 (3)	0.5686 (3)	0.3177 (3)	0.0331 (9)	
H6	−0.0632	0.6185	0.3277	0.040*	
C7	−0.0237 (3)	0.4886 (3)	0.3681 (3)	0.0356 (9)	
H7	−0.0690	0.4828	0.4127	0.043*	
C8	0.0399 (3)	0.4162 (3)	0.3531 (3)	0.0359 (9)	
H8	0.0380	0.3607	0.3880	0.043*	
C9	0.1059 (3)	0.4239 (2)	0.2882 (3)	0.0295 (8)	
H9	0.1482	0.3735	0.2781	0.035*	
C10	0.2855 (3)	0.5464 (2)	0.0178 (3)	0.0276 (8)	
C11	0.3816 (3)	0.5881 (3)	0.0352 (3)	0.0453 (11)	
H11	0.4136	0.6027	0.0995	0.054*	
C12	0.4320 (4)	0.6091 (4)	−0.0408 (4)	0.0664 (15)	
H12	0.4979	0.6378	−0.0276	0.080*	
C13	0.3872 (4)	0.5888 (3)	−0.1337 (3)	0.0533 (13)	
H13	0.4220	0.6032	−0.1850	0.064*	
C14	0.2922 (4)	0.5477 (3)	−0.1528 (3)	0.0485 (11)	
H14	0.2609	0.5336	−0.2174	0.058*	
C15	0.2416 (3)	0.5266 (3)	−0.0778 (3)	0.0377 (9)	
H15	0.1756	0.4981	−0.0919	0.045*	
C16	0.1143 (2)	0.2108 (2)	−0.0052 (2)	0.0218 (7)	
H16	0.1344	0.2502	0.0533	0.026*	
C17	0.1521 (3)	0.2565 (2)	−0.0890 (2)	0.0256 (8)	
H17A	0.1338	0.2183	−0.1476	0.031*	
H17B	0.1180	0.3168	−0.1024	0.031*	
C18	0.2684 (3)	0.2693 (2)	−0.0643 (3)	0.0308 (9)	
H18A	0.2863	0.3099	−0.0075	0.037*	
H18B	0.2927	0.2986	−0.1192	0.037*	
C19	0.3215 (3)	0.1774 (3)	−0.0425 (3)	0.0355 (9)	
H19A	0.3969	0.1869	−0.0231	0.043*	
H19B	0.3092	0.1392	−0.1015	0.043*	
C20	0.2811 (3)	0.1276 (3)	0.0380 (3)	0.0327 (9)	
H20A	0.3134	0.0663	0.0471	0.039*	
H20B	0.3018	0.1621	0.0990	0.039*	
C21	0.1647 (3)	0.1171 (2)	0.0165 (3)	0.0270 (8)	
H21A	0.1413	0.0894	0.0727	0.032*	
H21B	0.1439	0.0761	−0.0396	0.032*	
C22	−0.0561 (3)	0.1646 (2)	0.0476 (2)	0.0234 (8)	
H22	−0.0323	0.1001	0.0613	0.028*	
C23	−0.1710 (3)	0.1642 (3)	0.0064 (3)	0.0332 (9)	
H23A	−0.1847	0.1254	−0.0521	0.040*	
H23B	−0.1942	0.2272	−0.0123	0.040*	
C24	−0.2322 (3)	0.1273 (3)	0.0812 (3)	0.0368 (10)	
H24A	−0.3071	0.1303	0.0542	0.044*	
H24B	−0.2137	0.0623	0.0953	0.044*	
C25	−0.2087 (3)	0.1823 (3)	0.1736 (3)	0.0371 (9)	
H25A	−0.2462	0.1557	0.2216	0.045*	
H25B	−0.2330	0.2461	0.1607	0.045*	
C26	−0.0935 (3)	0.1823 (3)	0.2141 (3)	0.0402 (10)	
H26A	−0.0706	0.1190	0.2321	0.048*	
H26B	−0.0795	0.2203	0.2732	0.048*	
C27	−0.0316 (3)	0.2196 (3)	0.1407 (2)	0.0324 (9)	
H27A	−0.0494	0.2848	0.1271	0.039*	
H27B	0.0433	0.2158	0.1678	0.039*	

### Atomic displacement parameters (Å^2^)

**Table:**

	*U*^11^	*U*^22^	*U*^33^	*U*^12^	*U*^13^	*U*^23^
Sn	0.01965 (13)	0.01890 (12)	0.02124 (13)	0.00066 (11)	0.00070 (8)	0.00046 (11)
O1	0.0233 (14)	0.0203 (12)	0.0240 (13)	0.0026 (10)	−0.0033 (11)	−0.0020 (10)
O2	0.0238 (13)	0.0161 (13)	0.0292 (13)	0.0016 (9)	−0.0043 (10)	0.0021 (10)
O3	0.0284 (14)	0.0251 (13)	0.0266 (14)	−0.0012 (10)	−0.0089 (11)	0.0018 (10)
O4	0.0286 (14)	0.0193 (13)	0.0314 (14)	0.0034 (10)	−0.0025 (11)	0.0037 (10)
O5	0.0225 (13)	0.0177 (12)	0.0262 (13)	0.0014 (10)	−0.0020 (11)	−0.0027 (10)
O6	0.0217 (13)	0.0192 (12)	0.0201 (12)	0.0006 (10)	−0.0009 (10)	−0.0002 (10)
O7	0.0260 (16)	0.0237 (14)	0.0353 (16)	0.0013 (11)	−0.0007 (13)	−0.0018 (12)
N	0.0246 (17)	0.0186 (17)	0.0208 (17)	−0.0001 (12)	0.0042 (14)	−0.0004 (14)
C1	0.021 (2)	0.0227 (19)	0.0225 (19)	0.0008 (14)	0.0082 (16)	−0.0007 (15)
C2	0.0206 (19)	0.0233 (19)	0.0203 (18)	−0.0011 (14)	0.0060 (15)	−0.0010 (14)
C3	0.023 (2)	0.0193 (17)	0.0166 (16)	0.0008 (14)	0.0031 (14)	−0.0021 (13)
C4	0.0218 (17)	0.027 (2)	0.0175 (17)	−0.0008 (14)	−0.0009 (13)	−0.0022 (14)
C5	0.024 (2)	0.029 (2)	0.028 (2)	0.0001 (15)	−0.0010 (16)	0.0000 (15)
C6	0.021 (2)	0.045 (2)	0.033 (2)	−0.0008 (17)	0.0036 (17)	−0.0033 (18)
C7	0.029 (2)	0.048 (2)	0.032 (2)	−0.0127 (18)	0.0105 (17)	−0.0046 (19)
C8	0.036 (2)	0.038 (2)	0.034 (2)	−0.0119 (18)	0.0066 (19)	0.0024 (18)
C9	0.031 (2)	0.027 (2)	0.031 (2)	−0.0032 (16)	0.0058 (17)	−0.0017 (16)
C10	0.029 (2)	0.0265 (19)	0.029 (2)	0.0055 (15)	0.0077 (16)	0.0054 (15)
C11	0.030 (2)	0.070 (3)	0.036 (2)	−0.007 (2)	0.0053 (19)	0.008 (2)
C12	0.033 (3)	0.105 (4)	0.065 (4)	−0.011 (3)	0.017 (3)	0.019 (3)
C13	0.050 (3)	0.072 (3)	0.045 (3)	0.015 (2)	0.028 (2)	0.016 (2)
C14	0.069 (3)	0.048 (3)	0.031 (2)	0.004 (2)	0.015 (2)	0.0013 (19)
C15	0.048 (3)	0.035 (2)	0.033 (2)	−0.0038 (18)	0.0124 (19)	−0.0001 (18)
C16	0.0198 (19)	0.0236 (18)	0.0212 (18)	0.0027 (14)	0.0020 (15)	0.0001 (14)
C17	0.029 (2)	0.0263 (19)	0.0211 (19)	0.0022 (15)	0.0042 (16)	0.0009 (15)
C18	0.026 (2)	0.040 (2)	0.028 (2)	0.0013 (16)	0.0100 (17)	0.0019 (17)
C19	0.024 (2)	0.045 (2)	0.036 (2)	0.0081 (17)	0.0031 (17)	−0.0076 (18)
C20	0.027 (2)	0.035 (2)	0.034 (2)	0.0090 (16)	−0.0025 (17)	−0.0011 (17)
C21	0.028 (2)	0.0224 (19)	0.029 (2)	0.0033 (15)	0.0002 (16)	−0.0016 (15)
C22	0.027 (2)	0.0194 (18)	0.0237 (19)	0.0025 (14)	0.0054 (16)	0.0037 (14)
C23	0.030 (2)	0.042 (2)	0.028 (2)	−0.0047 (17)	0.0056 (17)	−0.0043 (17)
C24	0.026 (2)	0.049 (2)	0.037 (2)	−0.0064 (18)	0.0085 (18)	−0.0029 (19)
C25	0.032 (2)	0.047 (2)	0.035 (2)	−0.0018 (18)	0.0132 (18)	−0.0056 (19)
C26	0.038 (3)	0.058 (3)	0.026 (2)	−0.010 (2)	0.0104 (18)	−0.0021 (19)
C27	0.030 (2)	0.043 (2)	0.023 (2)	−0.0064 (17)	0.0038 (17)	−0.0043 (17)

### Geometric parameters (Å, °)

**Table:**

Sn—C10	2.121 (3)	C13—C14	1.368 (6)
Sn—C4	2.132 (3)	C13—H13	0.9500
Sn—O1	2.189 (2)	C14—C15	1.388 (5)
Sn—O2	2.229 (2)	C14—H14	0.9500
Sn—O5	2.269 (2)	C15—H15	0.9500
Sn—O7	2.416 (2)	C16—C17	1.522 (4)
Sn—O6	2.430 (2)	C16—C21	1.527 (4)
O1—C1	1.273 (4)	C16—H16	1.0000
O2—C2	1.273 (4)	C17—C18	1.517 (5)
O3—C2	1.225 (4)	C17—H17A	0.9900
O4—C1	1.247 (4)	C17—H17B	0.9900
O5—C3	1.259 (4)	C18—C19	1.518 (5)
O6—C3^i^	1.256 (4)	C18—H18A	0.9900
O7—H7A	0.886 (19)	C18—H18B	0.9900
O7—H7B	0.896 (19)	C19—C20	1.527 (5)
N—C16	1.500 (4)	C19—H19A	0.9900
N—C22	1.507 (4)	C19—H19B	0.9900
N—H1A	0.85 (4)	C20—C21	1.514 (5)
N—H1B	0.91 (4)	C20—H20A	0.9900
C1—C2	1.548 (5)	C20—H20B	0.9900
C3—O6^i^	1.256 (4)	C21—H21A	0.9900
C3—C3^i^	1.537 (7)	C21—H21B	0.9900
C4—C5	1.394 (5)	C22—C23	1.516 (5)
C4—C9	1.397 (5)	C22—C27	1.522 (5)
C5—C6	1.389 (5)	C22—H22	1.0000
C5—H5	0.9500	C23—C24	1.540 (5)
C6—C7	1.373 (5)	C23—H23A	0.9900
C6—H6	0.9500	C23—H23B	0.9900
C7—C8	1.390 (5)	C24—C25	1.514 (5)
C7—H7	0.9500	C24—H24A	0.9900
C8—C9	1.382 (5)	C24—H24B	0.9900
C8—H8	0.9500	C25—C26	1.518 (5)
C9—H9	0.9500	C25—H25A	0.9900
C10—C11	1.385 (5)	C25—H25B	0.9900
C10—C15	1.394 (5)	C26—C27	1.534 (5)
C11—C12	1.398 (6)	C26—H26A	0.9900
C11—H11	0.9500	C26—H26B	0.9900
C12—C13	1.366 (7)	C27—H27A	0.9900
C12—H12	0.9500	C27—H27B	0.9900
			
C10—Sn—C4	168.75 (13)	C14—C15—C10	121.4 (4)
C10—Sn—O1	97.50 (11)	C14—C15—H15	119.3
C4—Sn—O1	93.06 (10)	C10—C15—H15	119.3
C10—Sn—O2	92.53 (12)	N—C16—C17	109.4 (3)
C4—Sn—O2	94.18 (11)	N—C16—C21	111.8 (3)
O1—Sn—O2	73.55 (8)	C17—C16—C21	110.9 (3)
C10—Sn—O5	93.06 (12)	N—C16—H16	108.2
C4—Sn—O5	86.04 (10)	C17—C16—H16	108.2
O1—Sn—O5	74.35 (8)	C21—C16—H16	108.2
O2—Sn—O5	147.87 (8)	C18—C17—C16	109.9 (3)
C10—Sn—O7	86.30 (11)	C18—C17—H17A	109.7
C4—Sn—O7	88.01 (10)	C16—C17—H17A	109.7
O1—Sn—O7	140.57 (9)	C18—C17—H17B	109.7
O2—Sn—O7	67.06 (8)	C16—C17—H17B	109.7
O5—Sn—O7	144.90 (8)	H17A—C17—H17B	108.2
C10—Sn—O6	85.62 (11)	C17—C18—C19	110.2 (3)
C4—Sn—O6	83.54 (10)	C17—C18—H18A	109.6
O1—Sn—O6	144.21 (8)	C19—C18—H18A	109.6
O2—Sn—O6	142.15 (7)	C17—C18—H18B	109.6
O5—Sn—O6	69.87 (8)	C19—C18—H18B	109.6
O7—Sn—O6	75.09 (8)	H18A—C18—H18B	108.1
C1—O1—Sn	117.4 (2)	C18—C19—C20	111.1 (3)
C2—O2—Sn	117.3 (2)	C18—C19—H19A	109.4
C3—O5—Sn	119.8 (2)	C20—C19—H19A	109.4
C3^i^—O6—Sn	114.2 (2)	C18—C19—H19B	109.4
Sn—O7—H7A	104 (3)	C20—C19—H19B	109.4
Sn—O7—H7B	127 (3)	H19A—C19—H19B	108.0
H7A—O7—H7B	103 (4)	C21—C20—C19	112.4 (3)
C16—N—C22	118.5 (3)	C21—C20—H20A	109.1
C16—N—H1A	108 (2)	C19—C20—H20A	109.1
C22—N—H1A	105 (2)	C21—C20—H20B	109.1
C16—N—H1B	108 (2)	C19—C20—H20B	109.1
C22—N—H1B	109 (2)	H20A—C20—H20B	107.9
H1A—N—H1B	108 (3)	C20—C21—C16	109.6 (3)
O4—C1—O1	125.1 (3)	C20—C21—H21A	109.8
O4—C1—C2	118.2 (3)	C16—C21—H21A	109.8
O1—C1—C2	116.6 (3)	C20—C21—H21B	109.8
O3—C2—O2	126.6 (3)	C16—C21—H21B	109.8
O3—C2—C1	118.9 (3)	H21A—C21—H21B	108.2
O2—C2—C1	114.4 (3)	N—C22—C23	107.8 (3)
O6^i^—C3—O5	125.2 (3)	N—C22—C27	110.8 (3)
O6^i^—C3—C3^i^	117.5 (4)	C23—C22—C27	111.5 (3)
O5—C3—C3^i^	117.2 (4)	N—C22—H22	108.9
C5—C4—C9	117.9 (3)	C23—C22—H22	108.9
C5—C4—Sn	121.4 (2)	C27—C22—H22	108.9
C9—C4—Sn	120.6 (2)	C22—C23—C24	110.6 (3)
C6—C5—C4	121.3 (3)	C22—C23—H23A	109.5
C6—C5—H5	119.4	C24—C23—H23A	109.5
C4—C5—H5	119.4	C22—C23—H23B	109.5
C7—C6—C5	120.2 (3)	C24—C23—H23B	109.5
C7—C6—H6	119.9	H23A—C23—H23B	108.1
C5—C6—H6	119.9	C25—C24—C23	110.9 (3)
C6—C7—C8	119.4 (3)	C25—C24—H24A	109.5
C6—C7—H7	120.3	C23—C24—H24A	109.5
C8—C7—H7	120.3	C25—C24—H24B	109.5
C9—C8—C7	120.7 (3)	C23—C24—H24B	109.5
C9—C8—H8	119.7	H24A—C24—H24B	108.1
C7—C8—H8	119.7	C24—C25—C26	110.5 (3)
C8—C9—C4	120.6 (3)	C24—C25—H25A	109.5
C8—C9—H9	119.7	C26—C25—H25A	109.5
C4—C9—H9	119.7	C24—C25—H25B	109.5
C11—C10—C15	117.5 (3)	C26—C25—H25B	109.5
C11—C10—Sn	120.6 (3)	H25A—C25—H25B	108.1
C15—C10—Sn	121.9 (3)	C25—C26—C27	111.5 (3)
C10—C11—C12	120.7 (4)	C25—C26—H26A	109.3
C10—C11—H11	119.6	C27—C26—H26A	109.3
C12—C11—H11	119.6	C25—C26—H26B	109.3
C13—C12—C11	120.5 (4)	C27—C26—H26B	109.3
C13—C12—H12	119.7	H26A—C26—H26B	108.0
C11—C12—H12	119.7	C22—C27—C26	110.0 (3)
C12—C13—C14	119.8 (4)	C22—C27—H27A	109.7
C12—C13—H13	120.1	C26—C27—H27A	109.7
C14—C13—H13	120.1	C22—C27—H27B	109.7
C13—C14—C15	120.0 (4)	C26—C27—H27B	109.7
C13—C14—H14	120.0	H27A—C27—H27B	108.2
C15—C14—H14	120.0		

Symmetry codes: (i) −*x*, −*y*+1, −*z*.

### Hydrogen-bond geometry (Å, °)

**Table:**

*D*—H···*A*	*D*—H	H···*A*	*D*···*A*	*D*—H···*A*
O7—H7B···O4^ii^	0.90 (4)	1.77 (4)	2.663 (3)	175 (4)
N—H1A···O3^iii^	0.84 (4)	2.12 (3)	2.910 (4)	155 (3)
N—H1A···O4^iii^	0.84 (4)	2.37 (4)	2.986 (4)	130 (3)
N—H1B···O6^i^	0.91 (4)	2.08 (4)	2.960 (4)	164 (4)

Symmetry codes: (ii) −*x*+1/2, *y*+1/2, −*z*+1/2; (iii) *x*−1/2, −*y*+1/2, *z*−1/2; (i) −*x*, −*y*+1, −*z*.
